# FDG uptake in the cervical muscles after neck dissection: imaging features and postoperative natural course on ^18^F‑FDG‑PET/CT

**DOI:** 10.1007/s11604-024-01568-6

**Published:** 2024-04-25

**Authors:** Yukako Iritani, Hiroki Kato, Yo Kaneko, Takuma Ishihara, Tomohiro Ando, Masaya Kawaguchi, Hirofumi Shibata, Takenori Ogawa, Yoshifumi Noda, Fuminori Hyodo, Masayuki Matsuo

**Affiliations:** 1 Department of Radiology, Gifu University, 1-1 Yanagido, Gifu, 501-1194 Japan; 2 Innovative and Clinical Research Promotion Center, Gifu University Hospital, Gifu, Japan; 3 Department of Otolaryngology, Gifu University, Gifu, Japan; 4 Center for One Medicine Innovative Translational Research (COMIT), Institute for Advanced Study, Gifu University, Gifu, Japan

**Keywords:** PET, SUV, Head and neck cancer, Neck dissection, Cervical muscle

## Abstract

**Purpose:**

This study aimed to assess the imaging features and postoperative natural course of ^18^F-fluorodeoxyglucose (FDG) uptake in the cervical muscles after neck dissection.

**Materials and methods:**

This study included 83 patients who underwent preoperative and postoperative ^18^F-FDG-PET/CT and were diagnosed with head and neck malignancy after neck dissection. Postoperative ^18^F-FDG-PET/CT was performed within 5 years after neck dissection. Preoperative and postoperative FDG uptake of the trapezius, sternocleidomastoid, scalene, pectoralis major, and deltoid muscles was visually assessed. Increased postoperative uptake was visually defined as higher postoperative FDG uptake than the preoperative one in the corresponding muscle. The maximum standardized uptake value (SUVmax) was measured in cases with increased postoperative uptakes.

**Results:**

Increased postoperative uptakes were observed in 43 patients (52%). The trapezius (31/83, 37%), sternocleidomastoid (19/83, 23%), and scalene (12/83, 14%) muscles were involved, as opposed to the pectoralis major and deltoid muscles were not. Increased postoperative uptakes were observed on the dissected side in all 43 patients. Significant differences between SUVmax estimated from the mixed-effects model and postoperative months were observed in the trapezius muscle (Coefficient (*β*) = −0.038; 95% confidence interval (CI): [−0.047, −0.028]; *p* < 0.001) and sternocleidomastoid muscle (*β* =  −0.015; 95% CI: [−0.029, −0.001]; *p* = 0.046).

**Conclusions:**

Increased postoperative uptakes in the cervical muscles were observed on the dissected side in approximately half of the patients after neck dissection. The SUVmax in the trapezius and sternocleidomastoid muscles decreased after surgery over time.

## Introduction

Neck dissection is a surgical procedure to remove lymph nodes from the neck area, which is mainly performed for the purpose of cancer treatment. The lymph nodes in the neck are divided into seven levels by anatomic landmarks. Radical neck dissection (RND), which involves levels I–V, is the standard basic procedure for cervical lymphadenectomy. The modified radical neck dissection (MRND) removes all of the same lymph node groups as the RND but spares at least one of the nonlymphatic structures removed with the RND such as the sternocleidomastoid muscle, accessory nerve, or internal jugular vein. In contrast, selective neck dissection (SND) preserves one or more levels of lymph nodes, sparing most of these non-lymphatic structures.

In daily clinical practice, the accumulation of ^18^F-fluorodeoxyglucose (FDG) in the cervical muscles after neck dissection is common. The increased FDG uptake in the skeletal muscles after surgery is thought to be caused by denervation that stimulates the expression of glucose transporter (GLUT)-1 and GLUT-4 [1–4]. Therefore, ^18^F-FDG-PET/CT can be used to visualize muscle denervation and FDG uptake is associated with the severity and etiology of nerve injury [1, 3]. Several research studies have previously reported the frequency and distribution of postoperative FDG uptake in the cervical muscles after neck dissection [5–7]; however, to the best of our knowledge, no study has yet to investigate the postoperative natural course of FDG uptake. The accurate knowledge of natural course of FDG uptake in the cervical muscles after surgery may be important to differentiate from recurrence or metastasis and can avoid unnecessary additional examinations. Therefore, the present study aimed to assess the imaging features and postoperative natural course of FDG uptake in the cervical muscles after neck dissection.

## Materials and methods

### Patients

The present study was approved by the human research committee of the institutional review board of our hospital and complied with the guidelines of the Health Insurance Portability and Accountability Act of 1996. The requirement for informed consent was waived due to the retrospective nature of this study. We retrospectively reviewed our hospital’s electronic medical record system and identified 287 patients who underwent neck dissection between January 2017 and November 2022. Among them, 204 patients were excluded from this study due to the lack of preoperative ^18^F-FDG-PET/CT imaging alone (*n* = 70), lack of preoperative and postoperative ^18^F-FDG-PET/CT imaging (*n* = 69), lack of postoperative ^18^F-FDG-PET/CT imaging alone (*n* = 48), and different scanners between preoperative and postoperative ^18^F-FDG-PET/CT imaging (*n* = 17). In total, 83 patients were included.

#### ^18^F-FDG-PET/CT

All 83 patients underwent preoperative and postoperative whole-body ^18^F-FDG-PET/CT imaging from the skull to mid-thigh using ^18^F-FDG-PET/CT scanner with 16-row multidetector CT (Biograph Sensation 16; Siemens Medical Solutions, Malvern, PA, USA). Among them, total 178 postoperative ^18^F-FDG-PET/CT examinations (once in 33 patients, twice in 26, third in 14, fourth in five, fifth in 2, and seventh in three) were performed within 5 years after neck dissection. Briefly, after at least 4 h of fasting, patients received an intravenous injection of ^18^F-FDG (185 MBq). Blood glucose levels were checked in all patients before ^18^F-FDG injection, and no patient had a blood glucose level greater than 150 mg/dL. Approximately 60 min after ^18^F-FDG injection, CT and subsequent whole-body PET were performed.

Transverse images were reconstructed with 2 mm section thickness and no overlap. Oral or intravenous contrast agent was not used for CT. PET had an axial view of 16.2 cm per bed position with an intersectional gap of 3.75 mm in one bed position, which necessitated data acquisition in six or seven bed positions. Axial PET images were obtained using an imaging matrix of 256 × 256 and a field of view of 50 × 50 cm.

### Imaging assessment

Two radiologists with 19 and 3 years of post-training experience in nuclear medicine, who were blinded to clinical information, independently reviewed all ^18^F-FDG-PET/CT images on a commercially available DICOM viewer. The maximum intensity projection, axial PET, and axial fused PET/CT images were used for the evaluation. In cases with disagreement between the two reviewers, consensus was reached by discussion.

The reviewers qualitatively evaluated the FDG uptake in five muscles, namely the scalene muscle, which is related to cervical rotation and lateral flexion, and the trapezius, sternocleidomastoid, deltoid, and pectoralis major muscles, which attach to the clavicle. The reviewers visually compared the FDG uptake of these muscles between preoperative and postoperative images. When the postoperative FDG uptake was higher than the preoperative one in the corresponding muscle, the reviewers defined it as increased postoperative uptake. In the cases with increased postoperative uptake, the accumulation region within muscle was classified as diffuse (entire) or focal, and the maximum standardized uptake value (SUVmax) in the cervical muscles was measured. The number of postoperative examinations varied from one to seven times per patient, but the increased postoperative uptake was considered to be positive if it was seen even just once. In patients with increased postoperative uptake, who underwent multiple postoperative PET/CT examinations, the SUVmax was measured in each PET/CT examination.

### Statistical analysis

All statistical analyses were performed using the Statistical Package for Social Sciences Software (IBM SPSS Statistics for Windows, version 24.0. IBM Corp., Armonk, NY) and R version 4.3.3 (The R Foundation for Statistical Computing, Vienna, Austria). The Fisher’s exact test was performed to compare the frequency of increased postoperative uptake between patients with and without focal neck symptoms in the dissected areas, histological nodal metastasis, accessory nerve dissection, and radiation therapy. Interobserver variability for identifying increased postoperative uptake was assessed using kappa statistics. Because the SUVmax for each muscle was a repeat data with different measurement times, a mixed-effects model was used to confirm the change over time. Random intercepts were assumed for the mixed model. The association between SUVmax and time was confirmed by testing the null hypothesis that the regression coefficient is zero. A two-sided *p* value <0.05 was considered significant.

## Results

### Patients

Patients’ characteristics are summarized in Table 1. In total, 83 patients (67 males and 16 females; mean age, 65 years; age range, 33–84 years) were enrolled in this study. Patients were diagnosed with oropharyngeal cancer (*n* = 22), oral cancer (*n* = 19), hypopharyngeal cancer (*n* = 15), laryngeal cancer (*n* = 12), skin cancer (*n* = 5), parotid gland cancer (*n* = 4), thyroid cancer (*n* = 2), nasopharyngeal cancer (*n* = 1), esophageal cancer (*n* = 1), chondrosarcoma (*n* = 1), or anal cancer with cervical nodal metastasis (*n* = 1). The 19 cases with oral cancers included tongue (*n* = 13), floor of the mouth (*n* = 3), upper jaw (*n* = 2), and palatal (*n* = 1) cancers. Twenty-three patients underwent bilateral neck dissection, including bilateral SND (*n* = 16), bilateral MRND (*n* = 3), right MRND and left SND (*n* = 3), and right SND and left MRND (*n* = 1). Furthermore, 27 patients underwent right unilateral neck dissection, including SND (*n* = 18), MRND (*n* = 6), and RND (*n* = 3), and 33 patients underwent left unilateral neck dissection, including SND (*n* = 30) and MRND (*n* = 3). Postoperative focal neck symptoms in the dissected areas such as pain and stiffness were observed in 16 of 83 (19%) patients. Based on postoperative histological assessment, cervical nodal metastasis was observed in 63 of 83 (76%) patients. The accessory nerve was removed in 17 of 83 (20%) patients during neck dissection. Radiation therapy was performed in 38 of 83 (46%) patients.

**Table 1:** Patient characteristics

Number of patients	83
Age (years)	
Mean	65
Range	33–84
Gender	
Male	67
Female	16
Histoloigical subtypes of malignancies	
Oropharyngeal cancer	22
Oral cancer	19
Hypopharyngeal cancer	15
Laryngeal cancer	12
Skin cancer	5
Parotid gland cancer	4
Thyroid cancer	2
Nasopharyngeal cancer	1
Esophageal cancer	1
Chondrosarcoma	1
Anal cancer with cervical nodal metastasis	1
Neck dissection	
Bilateral	23
Bilateral SND	16
Bilateral MRND	3
Right MRND and left SND	3
Right SND and left MRND	1
RND	0
Right	27
SND	18
MRND	6
RND	3
Left	33
SND	30
MRND	3
RND	0
Accessory nerve dissection	
+	17
−	66

*SND* selective neck dissection, *MRND* modified radical neck dissection, *RND* radical neck dissection

### Imaging features

The frequency and distribution of increased postoperative uptake per patient are summarized in Table 2. In total, increased postoperative uptakes were observed in 43 of 83 patients (52%). Among the 83 patients included in the present study, the trapezius (31/83, 37%), sternocleidomastoid (19/83, 23%), and scalene (12/83, 14%) muscles were involved, as opposed to the pectoralis major and deltoid muscles were not. The increased postoperative uptake region in the trapezius and sternocleidomastoid muscles was diffuse in all cases, regardless of the presence or absence of accessory nerve dissection. Among nine cases with unilateral uptake in the scalene muscle, focal accumulation was observed in five cases, and diffuse in four. Among the remaining three cases with bilateral uptake in the scalene muscle, bilateral focal accumulation was observed in two cases, and focal and diffuse in one. The focal accumulation in the scalene muscle was observed within the anterior or middle scalene muscles.

**Table 2:** The frequency and distribution of increased postoperative uptake per patient

All muscles	43/83 (52)
Trapezius muscles	31/83 (37)
Sternocleidomastoid muscles	19/83 (23)
Scalene muscles	12/83 (14)
Pectoralis major muscles	0 (0)
Deltoid muscles	0 (0)

The data are expressed as raw patient numbers; numbers in parentheses are frequencies expressed as percentages

No significant difference was found in the frequency of increased postoperative uptake between patients with and without focal neck symptoms in the dissected areas (38% vs. 55%, *p* = 0.268) and histological nodal metastasis (56% vs. 40%, *p* = 0.225).

No significant difference was found in the frequency of increased postoperative uptake in the trapezius (41% vs. 36%, *p* = 0.800), sternocleidomastoid (29% vs. 21%, *p* = 0.523), and scalene (18% vs. 14%, *p* = 0.704) muscles between patients with and without accessory nerve dissection (Table 3).

**Table 3:** The frequency of increased postoperative uptake in patients with or without accessory nerve dissection

	Accessory nerve dissection+ (*n* = 17)	Accessory nerve dissection − (*n* = 66)	*p* value
Trapezius muscles	7 (41)	24 (36)	0.800
Sternocleidomastoid muscles	5 (29)	14 (21)	0.523
Scalene muscles	3 (18)	9 (14)	0.704

The data are expressed as raw patient numbers; numbers in parentheses are frequencies expressed as percentages

No significant difference was found in the frequency of increased postoperative uptake in the trapezius (34% vs. 40%, *p* = 0.653), sternocleidomastoid (18% vs. 27%, *p* = 0.438), and scalene (18% vs. 11%, *p* = 0.368) muscles between patients with and without radiation therapy (Table 4).

**Table 4:** The frequency of increased postoperative uptake in patients with or without radiation therapy

	Radiation therapy + (*n* = 38)	Radiation therapy − (*n* = 45)	*p* value
Trapezius muscles	13 (34)	18 (40)	0.653
Sternocleidomastoid muscles	7 (18)	12 (27)	0.438
Scalene muscles	7 (18)	5 (11)	0.368

The data are expressed as raw patient numbers; numbers in parentheses are frequencies expressed as percentages

Table 5 summarizes the laterality of increased postoperative uptake in the trapezius, sternocleidomastoid, and scalene muscles. Increased postoperative uptakes were observed on the dissected side in all patients. In patients who underwent unilateral neck dissection, increased postoperative uptakes were not observed on the non-dissected side. Among the 106 dissected necks, increased postoperative uptake was observed in 38% (40/106) of trapezius, 21% (22/106) of sternocleidomastoid, and 14% (15/106) of scalene muscles.

**Table 5:** The laterality of increased postoperative uptake in the cervical muscles

Uptake side	Dissection side		
	Both (*n* = 12/7/3)	Right (*n* = 5/6/4)	Left (*n* = 14/6/3)
Both (*n* = 9/3/3)	9/3/3	0/0/0	0/0/0
Right (*n* = 7/7/5)	2/1/1	5/6/4	0/0/0
Left (*n* = 15/9/4)	1/3/1	0/0/0	14/6/3

The data are expressed as raw patient numbers (*n* = trapezius muscle/sternocleidomastoid muscle/scalene muscle)

**Fig. 1:**
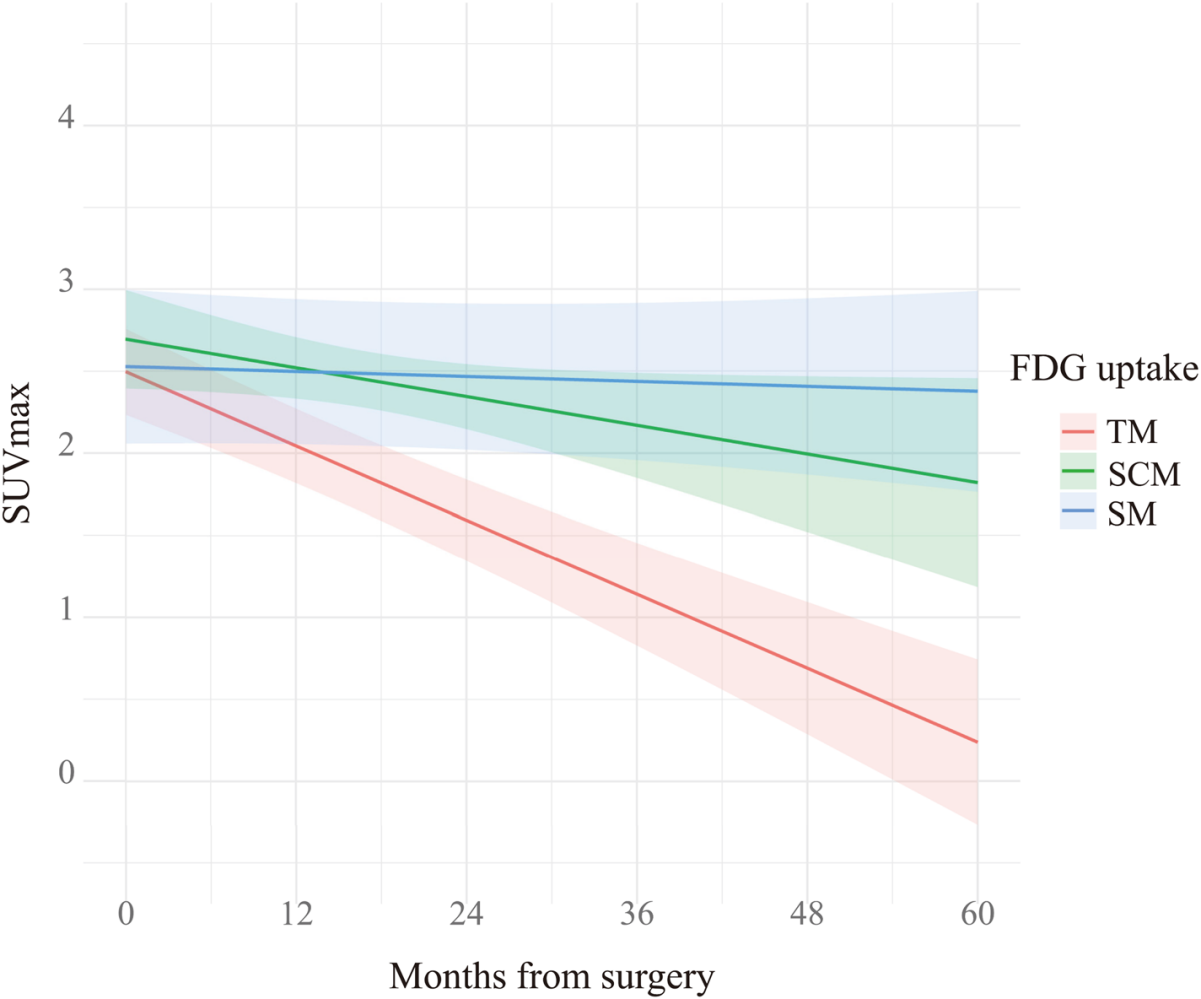
The association between SUVmax estimated from the mixed-effects model and postoperative months. The SUVmax in the trapezius and sternocleidomastoid muscles statistically decreased after surgery over time. The mean decreased SUVmax per month was 0.038 in the trapezius muscles and 0.015 in the sternocleidomastoid muscles. TM = trapezius muscle, SCM = sternocleidomastoid muscle, SM = scalene muscle

**Fig. 2:**
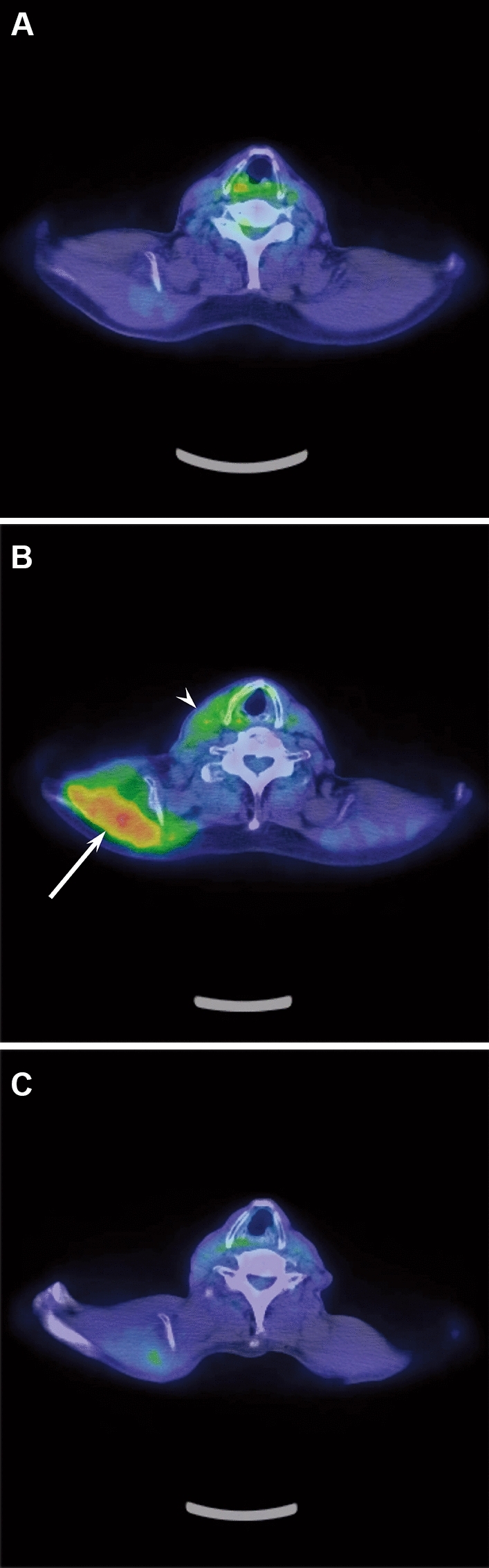
A 71-year-old man with right oropharyngeal cancer who underwent right unilateral modified radical neck dissection for levels I–V and right accessory nerve dissection. **a** Preoperative PET/CT image shows no accumulation of FDG in the cervical muscles. **b** Postoperative PET/CT image after 6 months of neck dissection shows FDG uptake in the right trapezius (SUVmax: 3.51) (arrow) and sternocleidomastoid (SUVmax: 2.25) (arrowhead) muscles on the dissected side. **c** Postoperative PET/CT image after 19 months of neck dissection shows decreased FDG uptake in the right trapezius and sternocleidomastoid muscles

The association between SUVmax estimated from the mixed-effects model and postoperative months is shown in Fig. 1 and Table 6. Significant differences between SUVmax estimated from the mixed-effects model and postoperative months were observed in the trapezius muscle (Coefficient (*β*) =  −0.038; 95% confidence interval (CI): [−0.047, −0.028]; *p* < 0.001) and sternocleidomastoid muscle (*β* =  −0.015; 95% CI: [−0.029, −0.001]; *p* = 0.046). The mean decreased SUVmax per month was 0.038 in the trapezius muscle and 0.015 in the sternocleidomastoid muscle (Fig. 2). However, no significant difference between SUVmax estimated from the mixed-effects model and postoperative months was observed in the scalene muscle (*β* =  −0.003; 95% CI: [−0.012, 0.007]; *p* = 0.609).

**Table 6:** The association between SUVmax estimated from the mixed-effects model and postoperative months

	Coefficient (*β*)	95% CI	*p* value
Trapezius muscles	−0.038	−0.047, −0.028	<0.001*
Sternocleidomastoid muscles	−0.015	−0.029, −0.001	0.046*
Scalene muscles	−0.003	−0.012, 0.007	0.609

*CI* confidence interval

* *p* value was less than 0.05

The Kappa values for the two reviewers exhibited excellent agreement regarding increased postoperative uptake in the scalene muscle (0.980), trapezius muscle (0.958), and sternocleidomastoid muscle (0.845).

## Discussion

In the present study, increased postoperative uptakes in the cervical muscles were observed in 52% of patients after neck dissection and were most frequently seen in the trapezius muscle (37%), followed by the sternocleidomastoid (23%) and scalene (14%) muscles. The SUVmax in the trapezius and sternocleidomastoid muscles decreased after surgery over time. A previous study investigating FDG uptake in the neck muscles for a time period of at least 6 months after neck dissection in patients with oral cancer showed that increased postoperative uptakes were frequently observed in the trapezius, sternocleidomastoid, and posterior neck muscles [5]. These findings are consistent with our results.

The physiologic or pathologic FDG uptake in the skeletal muscle is caused by various conditions, such as postprandial syndrome, use of insulin by patients with diabetes, vigorous muscle exercise, stress-induced muscle tension, spastic paresis, hyperventilation, muscle activity, hyperinsulinemia, hyperthyroidism, denervation, radiation therapy, rhabdomyolysis, polymyositis and dermatomyositis, eosinophilic myositis, and the presence of a tumor [8, 9]. Of these, denervation is believed to be one of the causes of FDG uptake in the skeletal muscles after surgery [1–4]. In neck dissection, the accessory nerves are often removed, and even if they are preserved, they can be easily injured by intraoperative traction [10]. The accessory nerve is a motor nerve that supplies the trapezius and sternocleidomastoid muscles; therefore, denervation of the trapezius and sternocleidomastoid muscles can be easily caused by accessory nerve damage during neck dissection [7]. Electromyography that was performed in the previous studies confirmed the increased FDG uptake in the denervated muscles with peripheral nerve injury [3, 7], whose mechanism relies on the increased expression of GLUT-1 and GLUT-4 [1]. Indeed, increased postoperative uptake was often observed in the trapezius and sternocleidomastoid muscles on the dissected side in the present study.

In the present study, the accumulations of FDG in the trapezius and sternocleidomastoid muscles did not always increase after accessory nerves resection. According to the majority of previous literatures, acute changes occur within a month following denervation, subacute changes between 12–20 months, and chronic changes after 12–20 months [11]. In this study, postoperative PET/CT was performed at least 56 days after neck dissection; therefore, no patients underwent postoperative PET/CT in acute phase of denervation. Meanwhile, in a previous study using rats, an increased FDG uptake in denervated muscles were observed just after the first 2 weeks, but no apparent uptake was observed at 5 weeks after denervation [2]. Thus, further investigation that performs PET/CT within a month after neck dissection may reveal the true relationship between increased postoperative uptakes and accessory nerves resection.

As a result of the present study assessing the association between SUVmax estimated from the mixed-effects model and postoperative months, the SUVmax in the trapezius and sternocleidomastoid muscles statistically decreased after surgery over time. In a previous study using male Sprague–Dawley rats, FDG uptakes in the denervated muscles were higher than those in the control muscles after 1 and 2 weeks of denervation, whereas no difference was found between the denervated and control muscles after 5, 8, or 10 weeks of denervation [2].

In the present study, increased FDG uptakes after neck dissection were also observed in the scalene muscle in addition to the trapezius and sternocleidomastoid muscles. The scalene muscle is innervated by the cervical plexus and brachial plexus. The cervical plexus is sometimes removed during radial and modified neck dissections, and damage to the cervical plexus during neck dissection may reduce sensorial perception in the neck [12]. The cervical plexus innervates the trapezius, sternocleidomastoid, scalene, infrahyoid, and geniohyoid muscles. However, the accumulation in the infrahyoid and geniohyoid muscles may be mild because these muscles have relatively small volume. In addition, increased postoperative uptakes in the scalene muscle may be caused by not only denervation due to cervical plexus transection during neck dissection but also vicarious load due to denervation of the trapezius and sternocleidomastoid muscles. Meanwhile, the brachial plexus is usually spared during neck dissection. Therefore, neck dissection does not generally affect many muscles innervated by the brachial plexus, including deltoid and pectoralis major muscles.

The present study demonstrated the absence of increased postoperative uptakes on the non-dissected side in patients who underwent unilateral neck dissection, as opposed to the trapezius, sternocleidomastoid, and posterior neck muscles that were observed on the non-dissected side in the previous study [5]. This inconsistency may be attributed to the different procedures followed for neck dissection and load for the cervical muscles on the non-dissected side. Among the 60 patients who underwent unilateral neck dissection, RND was performed in three patients in the present study. In contrast, RND was performed in all 24 patients with oral cancer who underwent unilateral neck dissection in the previous study [5]. In other words, increased postoperative uptake on the non-dissected side may occur after RND, but disappear after MRND/SND. Thus, further investigation is required to determine the postoperative uptakes on the non-dissected side.

In the present study, the frequency of increased postoperative uptake tended to be higher in patients with histological nodal metastasis than in those without, but no statistical difference was found. The presence of nodal metastasis extends the dissection areas and increases surgical invasiveness including nerve resection and traction. A previous study reported that postoperative muscle uptake tended to be increased with extensive surgical methods such as RND [5]. Ultimately, focal neck symptoms in the dissected areas, histological nodal metastasis, accessory nerve dissection, and radiation therapy were not independent factors for increased postoperative uptake; therefore, increased postoperative uptake may be caused by compositive factors.

This study has several limitations. First, the study was conducted at a single institution; thus, the present cohort included a relatively small number of cases. Second, the judgment for increased postoperative uptake was qualitatively determined without quantitative assessment. Third, postoperative FDG uptakes in patients with neck dissection were not compared with those without neck dissection (controls). Fourth, the patient cohort underwent several different types of surgical procedures. Hence, the uptake may be influenced by the extent of dissection and experience of the operator. Fifth, we did not use electromyography to assess the presence or absence of denervation.

In conclusion, increased postoperative uptake in the cervical muscles was observed on the dissected side in approximately half of our patients after neck dissection. The increased postoperative uptake was observed most commonly in the trapezius muscle, followed by the sternocleidomastoid and scalene muscles. In patients who underwent unilateral neck dissection, especially MRND and SND, increased postoperative uptakes were not observed on the non-dissected side. The SUVmax in the trapezius and sternocleidomastoid muscles decreased after surgery over time. The present study highlights that radiologists should be aware of the relatively high frequency of postoperative uptake in the cervical muscles after neck dissection and avoid a false recognition as recurrence or metastasis of head and neck cancer.
